# Relationships between migration and the fiscal sustainability of the pension system in China

**DOI:** 10.1371/journal.pone.0248138

**Published:** 2021-03-10

**Authors:** Haoyu Hu, Wei Wang, Dawei Feng, Hualei Yang

**Affiliations:** 1 School of Public Economics and Administration, Shanghai University of Finance and Economics, Shanghai, China; 2 Institute of Industrial Economics, Jiangxi University of Finance & Economics, Nanchang, China; 3 School of Public Administration, Zhongnan University of Economics & Law, Wuhan, China; Universidad de Murcia, SPAIN

## Abstract

There are a few existing studies on whether domestic migration improves China’s pension system’s fiscal sustainability in the context of rapid urbanization and industrialization. In this paper, we systematically investigate the impact of migration on the solvency of the worker’s old-age insurance for urban employees by constructing actuarial and econometric models. We use panel data from 2002 to 2018, collected from 31 provinces in China. The results show that the association between migration and the solvency of pensions is an inverted-U shape along the urbanization process. Further regional comparison showed that the above-stated inverted-U curve is more pronounced in the central and western regions. We also established that the number of participants and the contribution base are the main contributors to these results. Our conclusions are important for future population policies and public pension systems in China.

## Introduction

The fiscal sustainability of a pension system is crucial to the economic and social development of a country. It may directly affect the economic situation of a country and the government’s income [1] and change household saving behaviors and fertility decisions [2, 3]. As such, the solvency of pension systems has become the main focus of social security for governments worldwide, including China.

Since the year 2000, the Chinese population has been aging. Data show that as of the end of 2019, 12.6% of the total Chinese population was 65 years and above. This is an increase of 5.5% compared to the proportion in 2000 [4]. Meanwhile, life expectancy per capita has increased from 71.4 years in 2000 to 77.3 years by the end of 2019. It is expected to exceed 79 years by 2030 [5, 6]. In this regard, the Chinese pension system is facing the challenge of “breaking even with a slight surplus”. This has become a preferred topic in related academic discussions and public debates.

Many scholars use either the Lee-Carter, the stochastic projection, or other actuarial models to project possible income and expenditure gaps for pension systems when under pressure from an aging population and facing longevity risks. The majority of these scholars have projected severe repayment crises. For example, Zhao and Mi (2019) investigated the distribution of China’s pension gap over the next 50 years. They projected that without policy intervention, China’s pension gap will begin to manifest in 2017 and continue to expand until 2070 [7]. Liu (2014) projected that if the government’s financial subsidies for the pension system (i.e., implicit government debt) from 2010 to 2050 are discounted to the year 2010, the total amount may exceed 52.3 trillion yuan, which is equivalent to approximately 130% of the GDP in 2010 [8]. It is only a matter of time before the Chinese pension accounts show a gap between their revenue and expenditure, underlining the significance of research into the fiscal sustainability of the Chinese pension systems.

Another related phenomenon is the large-scale domestic migration in China. The last 40 years of reform and opening-up have incubated uneven regional economic development and unequal employment opportunities which have, in turn, led to domestic migration. Also, the relaxation of the household registration—the *Hu-Kou* system (registration of people living at a place other than their registered residence for over one month) has encouraged domestic migration. Statistical data show that from 1982 to 2019, the stock of Chinese people who have moved places has increased from 6.57 million to 236 million, meaning that, during that time, approximately 16.86% of the Chinese population has moved places [9]. In our investigation, we found substantial heterogeneity across the provinces (see S1 Fig). Shanghai, Beijing, and Tianjin had the top three migration rates of 2018, all exceeding 1.44%. We categorized them as net inflow areas. Guangxi, Henan, and Guizhou had the bottom three migration rates of 2018, with migration rates of 0.87%, 0.84%, and 0.79%, respectively. We categorized them as net outflow areas. We also found out that large-scale migration has a significant impact on the economic and social development of Chinese cities [10, 11].

However, few scholars have conducted systematic research on the impact of migration on the fiscal sustainability of the pension systems in China. Owing to high domestic migration, China has seen rapid urbanization and industrialization in the last 40 years. Therefore, investigating the association between migration and the fiscal sustainability of the pension systems in China will provide a valuable reference to other countries currently undergoing urbanization and facing the possibility of pension repayment crises.

Our work varies from previous studies in the following aspects: First, we employ a Chinese dataset, which is more representative of the social and economic situation of China; Second, we introduce an indicator of the urbanization rate and construct an actuarial model to study the effect of the number of participants and the contribution base; Third, to verify the reliability of our study, we build a rigorous econometric model to perform empirical tests on the theoretical propositions.

The remainder of this paper is arranged as follows: Section 2 introduces the background information and highlights some sections of relevant literature; Section 3 introduces the theoretical framework that is the basis of this study; Section 4 describes the econometric methodology and data; Section 5 reports the primary set of the estimation results; Section 6 provides further discussion and Section 7 presents the conclusions.

## Background and relevant literature

### The public pension system in China

The public pension system of China is comprised of worker’s old-age insurance for urban employees and urban and rural residents’ social pension insurance. The latter is mainly funded by fiscal subsidies which makes it more of a welfare system [7]. For this reason, we omitted it from our investigation. The worker’s old-age insurance for urban employees was formalized in 1997. It was introduced under the market economy context and initially covered employees from urban enterprises. After 2005, the coverage has gradually expanded to non-standard employees in informal sectors (e.g., own-account workers and employers). In 2014, it was merged with the pension for government and public institutions. Impressively, the public pension system has grown to cover nearly all urban employees. By the end of 2018, the total number of participants in this system reached 419 million, which is nearly four times the number of participants compared to when the system was established in 1997.

Meanwhile, as the number of retirees is increasing, the funds available to the pension system are running low despite all financial subsidies. S1 Table shows the balances of the public pension system from 2002 to 2018 [12]. As can be seen, the pension system has been spending more than it receives since 2009. The annual growth rate of the accumulative balance hit a new low of under 10% in 2016.

China’s public pension system is not centrally managed. Pension funds in most provinces are still mainly financed and managed by the provincial governments. As can be seen from S2 Table, if fiscal subsidies and interest income are excluded, more than 75% of the provinces will have an income-expenditure gap [13]. In 24 provinces, this issue is especially urgent, including three municipalities directly under the control of the central government (Tianjin, Chongqing, and Shanghai), industrialized provinces such as Jilin, Heilongjiang, and Liaoning. Clearly, the balance of the public pension fund depends on fiscal subsidies by the government in the near future to fill this gap.

### Previous literature

Scholars have proposed numerous solutions to adequately deal with the projected repayment crises facing public pension systems, as summarized in Table 1. In this section, we introduce some of these researches, specifically the pieces of research that relied on data from China and the organization for economic co-operation and development (OECD) countries.

**Table 1:** Key solutions regarding the fiscal sustainability of the pension system.

Solutions	Countries	Methodologies	Authors
Reducing the pension replacement rate	China	Actuarial model	Liu [8]
	The seven largest OECD countries (G7)	Projection and numerical analysis	Bongaarts [14]
	Japan	Overlapping generations (OLG) model and numerical analysis	Imrohoroglu, Kitao and Yamada [15]
Delaying the statutory retirement age	China	Projection and simulation, or actuarial model	Liu [8], Zeng [16], Zhao, Bai, Liu, and Hao [17]
	The seven largest OECD countries (G7)	Projection and numerical analysis	Bongaarts [14]
	Spain	OLG model and numerical analysis, or case-study-based calculations	Diaz-Gimenez and Diaz-Saavedra [18], Gaya, Carpio, Fabian, Goenechea and Fernandez [19]
	Japan	OLG model and numerical analysis	Imrohoroglu, Kitao and Yamada [15]
	The United Kingdom	Static model and numerical analysis	Blake and Mayhew [20]
Improving the fertility level	China	Projection and simulation, or actuarial model	Zeng [21], Zeng, Zhang and Liu [22]
	The seven largest OECD countries (G7)	Projection and numerical analysis	Bongaarts [14]
	——	OLG model and numerical analysis	Chen [23]
Perfecting the central pension transfer system	China	Actuarial model	Shi and Zeng [24]

Other scholars have elucidated the positive impacts brought about by certain conditions. For example, young and high-skilled migrants have been found to provide a higher net contribution to government finances [25–27]. Blake and Mayhew (2006) investigated the relationship between migration and the pension system of the UK and found that the stock of migrant workers have a positive impact on the financial balance of the UK pension system. The impact can be moderated by business cycle factors [20]. Pianese, Attias and Varga (2014) found out that migrant females in Italy have a higher fertility rate than local residents, which significantly improves the financial soundness of Italy’s pension system, at least for the medium-term [28]. Hansen and Schultz-Nielsen (2017) demonstrated that migrants from Western countries (more prosperous countries) have a positive impact on the solvency of the pension systems, while migrants from non-Western countries (poor countries) have a large negative one [29].

As can be seen from the above literature review, scholars have conducted many in-depth investigations on the impact of migration on the solvency of the pension systems in developed countries. However, little research has focused on developing countries. In this paper, we focus on China; we propose a research proposition verifiable by theoretical models.

## Theoretical framework

Based on an actuarial model constructed by previous scholars [22, 30], our model adds the contribution base as a factor in examining the impact of migration on the solvency of pension systems.

The four most critical indicators are defined as follows: within a region, the registered population refers to people who are registered in the region according to the law, regardless of their living status. The permanent population refers to people who have lived for at least one month in their current place. The migration rate is the ratio of the permanent population to the registered population. Provinces with a migration rate greater than one are labeled as net inflow areas, otherwise as net outflow areas. The solvency of the pension system is quantified by the ratio of pension income to expenditure.

### Model setting

In terms of pension expenditure, the number of employees who retired at stage *t*, who moved to region *i* at the age of *j* is set to *URPN*_*t*,*i*,*j*_. If the average age of retiring is *J* years, then one year before the retirement year of this cohort is *t*−(*j*−*J*). The wage level and planned distribution rate before retirement are ${\bar{w}}_{t-(j-J),i}$ and *s*_*t*−(*j*−*J*),*i*_, respectively. If the average pension growth rate of retired employees over the years is *g*, then the pension growth rate of employees retiring at stage *t* who moved into region *i* at the age of *j* can be given by *g*_*t*,*i*,*j*_ = (1+*g*)^(*j*−*J*)−1^. Therefore, the total pension expenditure *AC*_*t*_ is given by:

$$AC_{t}={\sum_{j=J}^{\infty }URPN_{t,i,j}{\bar{w}}_{t-(j-J),i}s_{t-(j-J),i}(1+g)}^{(j-J)-1}$$ (1)

In terms of pension income, we introduce the following variables for urban employees moving into region *i* during stage *t*. The total population, urban population, urban employed population, and the number of participants in the pension system are denoted as *N*_*t*,*i*_, *UN*_*t*,*i*_, *UEN*_*t*,*i*_, and *UEPN*_*t*,*i*_, respectively. The urbanization rate, registered unemployment rate, and urban employee pension coverage rate are denoted as *ur*_*t*,*i*_, *urur*_*t*,*i*_, and *crp*_*t*,*i*_, respectively. The contribution base and contribution rate are denoted as ${\bar{w}}_{t,i}$ and *R*_*t*,*i*_, respectively. Therefore, the total pension income *AI*_*t*,*i*_ is given by:

$$AI_{t,i}=N_{t,i}ur_{t,i}(1-urur_{t,i})crp_{t,i}{\bar{w}}_{t,i}R_{t,i}$$ (2)

In general, migrants mainly move to areas that engage in non-agricultural employment activities. According to the formula for calculating the urbanization rate, migration can increase the urbanization rate of the migrant-receiving areas. In the case of regional population growth being the only consideration; and the urban population moving into region *i* (from outside region *i*) during stage *t* set to *MUN*_*t*,*i*_, and the total urban population moving within region *i* set to *NUN*_*t*,*i*_, then, the urbanization rate can be expressed as:

$$ur_{t,i}=\frac{UN_{t-1,i}+NUN_{t,i}+MUN_{t,i}}{N_{t-1,i}+MUN_{t,i}}$$ (3)

There are two relationships between variables in the above equation. That is *N*_*t*−1,*i*_+*MUN*_*t*,*i*_ = *N*_*t*,*i*_ and *UN*_*t*−1,*i*_+*NUN*_*t*,*i*_+*MUN*_*t*,*i*_ = *UN*_*t*,*i*_. Considering that the urbanization rate shows S-shaped characteristics [31], we have $\frac{\partial ur_{t,i}}{\partial MUN_{t,i}}>0$ and $\frac{\partial^{2}ur_{t,i}}{\partial^{2}MUN_{t,i}}<0$, which means that the population moving into region *i* increases with an upper bound. i.e., $\frac{\partial UN_{t,i}}{\partial MUN_{t,i}}>0$ and $\frac{\partial^{2}UN_{t,i}}{\partial^{2}MUN_{t,i}}>0$.

Next, we define *w*_*t*,*em*_, *w*_*t*,*i*_, and *w*_*t*,*im*_ as the wages of three different subpopulations of migration in region *i*. Specifically, *w*_*t*,*em*_ denotes migrants from only outside region *i*, *w*_*t*,*i*_ denotes the total migration within region *i*, and *w*_*t*,*im*_ denotes the total urban population. Considering that migration is mostly driven by economic factors, usually *w*_*t*,*em*_<*w*_*t*,*im*_<*w*_*t*,*i*_. If the registered unemployment rate and the contribution rate remain unchanged, we have (1−*urur*_*t*,*i*_)*crp*_*t*,*i*_ ≡ *K*. By setting the natural growth rate of the wage level to *m*_*t*_, the average contribution base in the migrant-receiving region *i* during stage *t* can be expressed as:

$${\bar{w}}_{t,i}=K(\frac{MUN_{t,i}}{UEPN_{t,i}}w_{t,em}+\frac{UN_{t-1,i}}{UEPN_{t,i}}w_{t,i}+\frac{MUN_{t,i}}{UEPN_{t,i}}w_{t,ir})m_{t}$$ (4)

Specifically, *UEPN*_*t*,*i*_ = *N*_*t*,*i*_*ur*_*t*,*i*_(1−*urur*_*t*,*i*_)*crp*_*t*,*i*_. Thus, with *w*_*t*,*em*_<*w*_*t*,*im*_<*w*_*t*,*i*_, then $\frac{\partial {\bar{w}}_{t,i}}{\partial MUN_{t,i}}<0$. By substituting Eqs (3) and (4) into (2), if the planned distribution rate remains constant, that is *R*_*t*,*i*_ ≡ *R*, the pension income *AI*_*t*,*i*_ for the migrant-receiving region *i* during stage *t* can be expressed as:

$$AI_{t,i}=UN_{t,i}(MUN_{t,i}){\bar{w}}_{t,i}(MUN_{t,i})KR$$ (5)

Compared to the traditional actuarial model, the critical difference between Eqs (5) and (2) is that the urbanization rate and contribution base are no longer given exogenously. Instead, our model is mostly affected by *MUN*_*t*,*i*_, the urban population that moves into region *i* (from outside region *i*) during stage *t*. Referring to the definition of pension solvency by Wang [32], we set the accumulative balance at the end of the previous period to *F*_*t*−1_. We then approximated the pension solvency *ppc*_*t*,*i*_ by adding *F*_*t*−1_ to the current pension income and dividing the result by the current pension expenditure. The expression is:

$$ppc_{t,i}=\frac{F_{t-1}+AI_{t,i}}{AC_{t}}$$ (6)

### A solution of the model

Pension expenditure is mainly affected by historical wage levels and the population that has retired. Although the contribution base can also affect pension expenditure, especially pension expenditure on newly retired employees, this subgroup accounts for a relatively small number of the total number of retired employees and is therefore negligible. Also, the accumulated balance of pension systems at the end of a previous period can be affected by the current migration, but the impact of this migration on pension expenditure is also negligible.

In terms of pension income, migration has a significant impact. It mainly affects two aspects, the contribution base and the number of participants. If $\frac{\partial {\bar{w}}_{t,i}}{\partial MUN_{t,i}}<0$ and $\frac{\partial UN_{t,i}}{\partial MUN_{t,i}}>0$, it means that the effect of the contribution base is negative, and the effect of the number of participants is positive. Differentiating Eq (6), we have

$$\frac{\partial ppc_{t,i}}{\partial MUN_{t,i}}\approx \frac{1}{AC_{t}}\frac{\partial AI_{t,i}}{\partial MUN_{t,i}}=\frac{KR}{AC_{t}}(UN_{t,i}\frac{\partial {\bar{w}}_{t,i}}{\partial MUN_{t,i}}+\frac{\partial UN_{t,i}}{\partial MUN_{t,i}}{\bar{w}}_{t,i})m_{t}$$ (7)

With *K*, *R*, *AC*_*t*_ and *m*_*t*_ being positive, whether migration can improve pension solvency will mainly depend on the sign of $UN_{t,i}\frac{\partial {\bar{w}}_{t,i}}{\partial MUN_{t,i}}+\frac{\partial UN_{t,i}}{\partial MUN_{t,i}}{\bar{w}}_{t,i}$. In other words, it will mainly depend on the relative size of the contribution base and the number of participants. By setting $Z=UN_{t,i}\frac{\partial {\bar{w}}_{t,i}}{\partial MUN_{t,i}}+\frac{\partial UN_{t,i}}{\partial MUN_{t,i}}{\bar{w}}_{t,i}$, if *Z*>0, then $\frac{\frac{\partial UN_{t,i}}{\partial MUN_{t,i}}}{UN_{t,i}}>-\frac{\frac{\partial {\bar{w}}_{t,i}}{\partial MUN_{t,i}}}{{\bar{w}}_{t,i}}$, which means that when the growth rate of the urban population is higher than the growth rate of the contribution base, migration will improve the solvency of pensions in this area [20, 33]. The improvement is due to the dominance of the effect caused by an increase in the number of participants. Conversely, when the effect of contribution base dominates, migration will reduce the pension solvency of the area [34].

What accelerates the urban population growth rate? What decelerates the wage growth rate? According to some scholars [31, 35], the urbanization process can be divided into three stages: early stage, middle stage, and late stage. In the early stage, the urbanization rate is usually low (approximately 30%). At this time, the dominating rural population is engaged in agricultural activities with low productivity, and the urbanization process is slow. In the middle stage (approximately 45% urbanization rate), agricultural productivity increases rapidly. With the gradual acceleration of industrialization, the rural labor force becomes excessive, and they begin to move to urban areas causing the urbanization rate to steadily increase. In the late stage, with the excess labor force decreasing in rural areas, and the labor difference between urban and rural areas diminishing, migration from rural to urban areas becomes relatively low. It gradually tends to stagnate, causing the urbanization rate to rise slowly.

Specifically, in the early stage of urbanization, due to the relatively low growth rate of the urban population and wages, the effect of the contribution base and the number of participants is not pronounced. In the middle stage of urbanization, due to the higher growth rate of the urban population, the supply of labor force is higher than demand. The wage growth rate is relatively low and inelastic owing to the impact(s) of informal employment, wage rigidity in the urbanization process, and the impact(s) of early industrialization. When the urban population growth rate is higher than that of the contribution base, the effect of the number of participants becomes more pronounced. This is because in the late urbanization stage, urban population growth slows down, and the whole society enters a post-industrial period. The demand for labor surpasses supply, and the market continues to improve. As formal employment opportunities increase and the sensitivity of wage adjustment strengthens, wages gradually become more flexible with a higher growth rate. When the urban population growth rate is less than that of the contribution base, the effect of the contribution base begins to show. As such, we propose the following theoretical proposition.

**Proposition.** In the early stage of urbanization, migration may not significantly impact the solvency of the pension system in migrant-receiving areas. In the middle stage of urbanization, migration has a significant positive impact on the solvency of the pension system in migrant-receiving areas. In the late stage of urbanization, migration may harm the solvency of the pension system in migrant-receiving areas.

Further investigation shows that in the early urbanization stage, due to the relatively weak effect of the contribution base and the participation rate, migration may not significantly impact the solvency of the pension system in migrant-receiving areas. In the middle urbanization stage, when the urbanization rate is steadily increasing, migration may significantly increase the solvency of the pension system in migrant-receiving areas owing to the dominance of the participation rate effect. In the late urbanization stage, migration can harm the solvency of the pension system in migrant-receiving areas (for a detailed description, see S2 Fig).

## Empirical framework

### Econometric methodology

1. According to the results of the above theoretical model, we find that under different urbanization stages, the effect of the number of participants and the contribution base is different, which leads to different impacts of migration on the solvency of the pension system. Therefore, the baseline model is set as follows:

$$\begin{array}{l} Solvency_{j,t}=\alpha_{0}+\alpha_{1}Migration_{j,t}+\alpha_{2}Migration_{j,t}*Urbanization_{j,t}+\\ \alpha_{3}Migration_{j,t}*Urbanization_{j,t}{}^{2}+\delta X_{j,t}+\eta_{j}+\varepsilon_{j,t} \end{array}$$ (8)

In Eq (8), *j* and *t* represent province and time, respectively. *Solvency*_*j*,*t*_ is the dependent variable, representing the pension solvency of province *j* in the year *t*. *Migration*_*j*,*t*_ is a core explanatory variable, representing the rate of migration of province *j* in the year *t*. Due to the non-linearity correlation between migration and pension solvency in the migrant-receiving areas at different urbanization stages, we introduce *Migration*_*j*,t_*Urbanization_*j*,*t*_ and *Migration*_*j*,t_*Urbanization_*j*,*t*_^2^ into the model. We focus on the significance and change of the coefficients *α*_2_ and *α*_3_. Variable *X*_*j*,*t*_ is a vector of control variables. Referring to Han’s practices (2013), we select the old-age dependency ratio, economic development level, pension benefits of retirees, the proportion of medical insurance fund expenditure, and maternity insurance fund expenditure as control variables [33]. Furthermore, we introduce *η*_*j*_ as the fixed effects of provinces to help control the impact of noisy factors that do not change over time in the same province (e.g., policies, institutions, and cultural norms). The goal is to minimize the estimation bias caused by missing variables. We use the variable *ε*_*j*,*t*_ for the random error term.

To better demonstrate the effectiveness of the number of participants and the contribution base, we constructed the following two models to test the impact mechanisms:

$$\begin{array}{l} U_{j,t}=\beta_{0}+\beta_{1}Migration_{j,t}+\beta_{2}Migration_{j,t}*Urbanization_{j,t}+\\ \beta_{3}Migration_{j,t}*Urbanization_{j,t}{}^{2}+\delta X_{j,t}+\eta_{j}+\varepsilon_{j,t} \end{array}$$ (9)

$$\begin{array}{l} V_{j,t}=\beta_{0}+\beta_{1}Migration_{j,t}+\beta_{2}Migration_{j,t}*Urbanization_{j,t}+\\ \beta_{3}Migration_{j,t}*Urbanization_{j,t}{}^{2}+\delta X_{j,t}+\eta_{j}+\varepsilon_{j,t} \end{array}$$ (10)

*U*_*j*,*t*_ and *V*_*j*,*t*_ are dependent variables representing the number of pension insurance participants and contribution base (measured by the average wage of employees), respectively. The definition of other variables is consistent with Eq (8). In Eqs (9) and (10), if the results of the interaction terms are different, it will be due to the impact of the two mechanisms and the non-linearity correlation between migration and pension solvency.

### Data and variables

The data used in this study are the panel data of all 31 provinces of China from 2002 to 2018. The original data are from the *China Statistical Yearbook*, *China’s Population and Employment Statistics Yearbook*, and *China Labor Statistics Yearbook*. We interpolated the available data to get some of the missing data. Table 2 reports the descriptive statistical results of the main variables. We find that the core variables, such as pension solvency and migration rate, have large variance across different regions. For example, the average value of national pension solvency is 2.380, with a range of 0.492 to 6.692. The average of the eastern region is 2.552, which is significantly higher than that of the central and western regions.

**Table 2:** Summary statistics for main variables.

Variables	Definition	Nationwide	Eastern	Central	Western
**Dependent variables**					
Pension solvency	(Accumulative pension balances + current pension revenues) / current pension expenses	2.380 (0.898)	2.552 (1.201)	2.197 (0.634)	2.345 (0.670)
**Independent variables**					
Migration rate	Share of total permanent population in registered population	1.033 (0.154)	1.147 (0.201)	0.965 (0.057)	0.976 (0.064)
Urbanization rate	Share of urban permanent population in total permanent population	0.507 (0.152)	0.633 (0.147)	0.471 (0.083)	0.415 (0.107)
Old-age dependency ratio	The proportion of active workers to pensioners	12.73 (2.869)	13.51 (2.690)	12.80 (2.326)	11.98 (3.158)
Economic development level	GDP per capita (10 thousand, CNY)	3.159 (2.403)	4.650 (2.908)	2.449 (1.470)	2.266 (1.610)
Pension benefits	Average pension benefits of retirees (10 thousand, CNY)	1.978 (1.201)	2.110 (1.102)	1.673 (0.936)	2.061 (1.398)
Medical insurance	Share of medical insurance fund expenditure in GDP	0.884 (0.579)	0.979 (0.649)	0.649 (0.429)	0.953 (0.556)
Maternity insurance	Share of maternity insurance fund expenditure in GDP (t-1, ‱)	3.355 (3.677)	4.859 (4.886)	1.804 (1.514)	3.010 (2.798)
**Mediating variables**					
Number of participants	Number of participants of pension insurance (10 million)	0.642 (0.662)	1.066 (0.897)	0.589 (0.231)	0.290 (0.269)
Contribution base	The average wage of active workers (10 thousand, CNY)	4.090 (2.497)	4.712 (2.886)	3.417 (1.903)	3.970 (2.333)

**Notes:** In this table, we have reported the mean value and standard deviation (presented in parentheses).

Moreover, the data show that the average national migration rate is 1.033. The average migration rates of the central and western regions are lower than 1, and the average of the eastern region is higher than 1. The results indicate that most eastern provinces are net inflow areas. Other control variables, such as the old-age dependency ratio, GDP per capita, and pension benefits, also have significant variances and noticeable regional differences. We assert that all the data meet the requirements of statistical and econometrical analysis.

## Empirical analysis

### Baseline estimates

Table 3 reports the baseline estimates of the effect of migration on pension solvency. From Column (1), we can find that as the urbanization rate continues to increase, the impact of migration on the solvency of the pension system will show an inverted-U curve. This observation verifies the above theoretical proposition. Specifically, there is an enormous surplus of the labor force in rural areas in the middle stage of urbanization. The excess labor force leads to inelastic wages with relatively low growth. Consequently, most of the rural labor force begins to move to urban areas, causing the urbanization rate to increase rapidly. After the internal urban population growth rate exceeds the wage growth rate, the effect of the number of participants begins to dominate. With the rapid increase in the number of urban employees participating in pension insurance, contribution to the pension fund increases sharply, which improves the solvency of the pension systems in migrant-receiving areas [20, 33].

**Table 3:** Migration and the solvency of public pensions (FE estimation).

Variables	Dependent variable: Pension solvency		
	(1)	(2)	(3)
Migration rate	-13.571***		
	(3.362)		
Migration rate * Urbanization rate	28.600***		
	(5.500)		
Migration rate * Urbanization rate ^2^	-14.782***		
	(3.123)		
Urbanization rate	-15.654***		
	(5.700)		
Migration rate * Year_2002		-0.689***	-0.696***
		(0.100)	(0.089)
Migration rate * Year_2004		-0.327***	
		(0.050)	
Migration rate * Year_2005			-0.096*
			(0.047)
Migration rate * Year_2006		0.034	
		(0.047)	
Migration rate * Year_2008		0.270***	0.293***
		(0.047)	(0.051)
Migration rate * Year_2010		0.218***	
		(0.060)	
Migration rate * Year_2011			0.313***
			(0.086)
Migration rate * Year_2012		0.171***	
		(0.057)	
Migration rate * Year_2014		-0.042	0.028
		(0.070)	(0.062)
Migration rate * Year_2016		-0.316***	
		(0.062)	
Migration rate * Year_2017			-0.138
			(0.091)
Migration rate * Year_2018		-0.377***	
		(0.119)	
Other controls	✓	✓	✓
Provincial fixed effects	✓	✓	✓
Observations	527	527	527
Adj R^2^	0.741	0.740	0.724

Notes

***, **, and * indicate significance at 1%, 5%, and 10%, respectively. Robust standard errors with a cluster at the province level are presented in parentheses. Other controls include old-age dependency ratio, economic development level, pension benefits, medical insurance, and maternity insurance.

However, affected by the Lewis turning point, the industrialization process gradually comes to an end, and the entire society enters the late urbanization stage. At this time, the dual structure of urban-rural disappears and the supply of excess rural labor force is depleted [36]. The urban population growth rate slowly declines, wages become relatively flexible and adjustable, and the growth rate of migrant wages exceeds that of urban population growth. Hence, the effect of the contribution base becomes the more dominant. The massive influx of population directly causes a rapid decline in the contribution base [34, 37], which in turn reduces the solvency of the pension system in the migrant-receiving areas.

Since China was in the middle stage of urbanization in 1997 when it formally established the pension system, the early urbanization stage proposition is difficult to verify. Data shows that China’s urbanization rate exceeded 30% in 1996 and grew to 59.58% by 2018. This means that China was in the middle stage of urbanization in the past two decades and gradually transitioned to the late urbanization stage.

**Fig 1:**
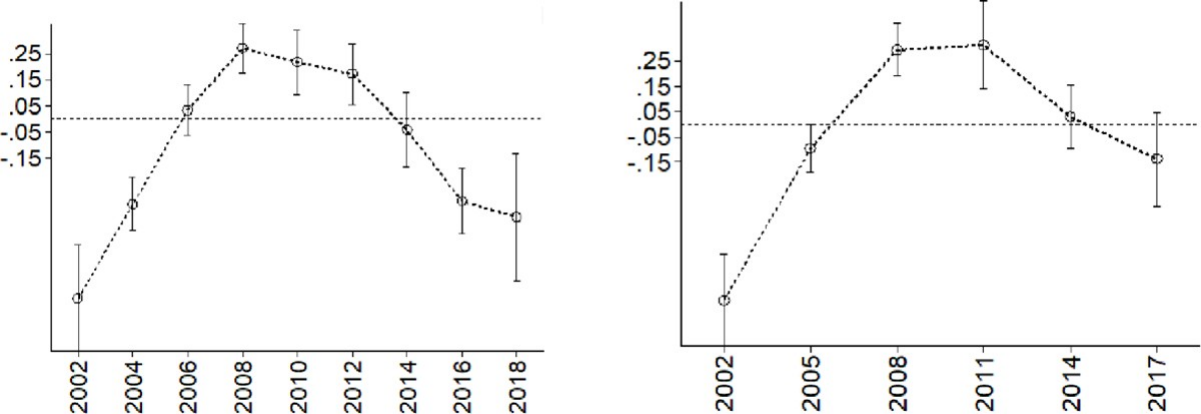
The dynamic impact of migration on the solvency of public pensions.

To precisely reveal the dynamic changes in migration on the pension solvency of the migrant-receiving areas, we combined China’s urbanization growth rates over the last 20 years (see S3 Fig) and incorporated the interaction terms of the migration rates and time into the model [38]. The corresponding regression results are reported in Columns (2)–(3) of Table 3. A comparison of the impacts in different years is illustrated in Fig 1. The solid line shows the marginal effect of migration, and the dashed line shows the 95% confidence interval. Specifically, between 2002 and 2005, the marginal effects were significantly negative at the 10% significance level. Between 2008 and 2012, the marginal effects became significantly positive at the 1% significance level. From 2016, the significant negative correlation started to show up again. Overall, it can be seen that the impact of migration on the solvency of pensions shows an inverted-U shape with an increase in the urbanization rate.

### Estimates correcting for endogeneity

Although the above estimation results are quite robust, the endogeneity problem due to missing variables and reverse causality should be considered because it will cause bias in the estimated results. The missing variables mainly refer to those unobservable variables, such as the residence permit system, that potentially affect how migration impacts pension solvency. These variables are difficult to quantify accurately. Reverse causality refers to the opposite effect of pension solvency applied on migration. For example, some areas with strong pension solvency may choose to tighten migration policies, by methods such as introducing some restrictions to elderly migrant workers.

In this study, we applied two methods to deal with potential endogeneity problems. The first method uses lagged variables. We use the lagged migration rate to replace the original independent variables. This will weaken the estimation bias caused by reverse causality to a certain extent. The results are presented in Table 4. In Columns (1)–(3), we took 1, 2, and 3 periods lagging of the migration rates and put them into the regression equation. We found that the new results are consistent with the previous results.

**Table 4:** Endogeneity tests (FE and 2SLS estimation results).

Variables	Dependent variable: Pension solvency			
	(1)	(2)	(3)	(4)
Migration rate_t-1_	-13.049***			
	(4.037)			
Migration rate_t-1_* Urbanization rate	28.581***			
	(5.786)			
Migration rate_t-1_* Urbanization rate ^2^	-15.548***			
	(3.660)			
Migration rate_t-2_		-11.843***		
		(3.778)		
Migration rate_t-2_* Urbanization rate		26.359***		
		(5.803)		
Migration rate_t-2_* Urbanization rate ^2^		-14.403***		
		(4.657)		
Migration rate_t-3_			-10.631***	
			(3.639)	
Migration rate_t-3_* Urbanization rate			22.594***	
			(6.908)	
Migration rate_t-3_* Urbanization rate ^2^			-11.672	
			(7.567)	
Migration rate				18.556
				(16.088)
Migration rate * Urbanization rate				16.541***
				(5.769)
Migration rate * Urbanization rate ^2^				-17.764***
				(6.693)
Urbanization rate	-15.833***	-15.244**	-15.039**	19.518
	(6.747)	(6.592)	(6.726)	(17.524)
Other controls	✓	✓	✓	✓
Provincial fixed effects	✓	✓	✓	✓
Observations	496	465	434	527
Adj R^2^	0.755	0.771	0.789	0.728
Weak IV test				75.0
				[9.08]
IV identification test				20.09
				<0.000>

Notes

***, **, and * indicate significance at 1%, 5%, and 10%, respectively. Robust standard errors with a cluster at the province level are presented in parentheses. Other controls include old-age dependency ratio, economic development level, pension benefits, medical insurance, and maternity insurance.

The second method is the instrumental variable (IV) method. In this method, we selected regional relative wage as the IV. Regional relative wage is the ratio of the average wages of employees in each province to the national average of employee wages. To select this variable, we first considered its relevance, since migration decisions largely depend on economic factors, an area with a higher average wage tends to be more attractive to the labor force, resulting in higher migration into the area. Second, we considered its heterogeneity, there is no apparent two-way causal relationship between relative wages and current pension solvency. Therefore, the regional relative wage is a viable IV that can be used in this study.

Column (4) of Table 4 reports the estimated results of the IV. First, we look at the results of the weak IV test. The Cragg-Donald Wald F statistic is 75.0, which is far higher than the corresponding critical value under a tolerance of 10% provided by Stock and Yogo [39]. This indicates that there is no weak IV. Second, according to the IV identifiable test results, the Kleibergen-Paap rk LM statistic is 97.186, and the p-value is 0.000. It rejects the null hypothesis that IV is not identifiable at the 1% significance level. Finally, based on the estimated results of the IV, we find that even considering endogeneity bias, at the different stages of urbanization, there is still a significant inverted-U relationship between migration and pension solvency in migrant-receiving areas.

## Further discussion

### Regional differences

So far, we have systematically investigated the impact of migration on the pension solvency in the migrant-receiving areas of China. Here, considering the imbalances in economic development and urbanization levels over different regions, we further investigated the heterogeneous effects by region. The results are shown in Table 5.

**Table 5:** Heterogeneous effects by region (FE estimation results).

Variables	Dependent variable: Pension solvency	
	Eastern region	The central and western region
	(1)	(2)
Migration rate	2.581	-11.836**
	(15.229)	(5.162)
Migration rate * Urbanization rate	7.656	28.843*
	(23.572)	(13.746)
Migration rate * Urbanization rate ^2^	-11.705	-15.269*
	(7.337)	(7.858)
Urbanization rate	5.888	-9.818
	(18.263)	(12.993)
Other controls	✓	✓
Provincial fixed effects	✓	✓
Observations	187	340
Adj R^2^	0.867	0.546

Notes

***, **, and * indicate significance at 1%, 5%, and 10%, respectively. Robust standard errors with a cluster at the province level are presented in parentheses. Other controls include the elderly dependency ratio, economic development, pension benefits, medical insurance, and maternity insurance.

From Columns (1)–(3), we find that the inverted-U relationship between migration and the solvency of pension systems in migrant-receiving areas is mainly pronounced in the central and western regions. This phenomenon may be related to the fact that, between 2002 and 2018, the central and western regions experienced two critical periods, the middle stage of urbanization and transition to the late stage.

Statistical data shows that the urbanization rates in the central and western regions in 2002 were 37.19% and 30.28%. By the end of 2018, they increased to 56.85% and 52.26%, respectively. Therefore, the central and western regions are going through the middle urbanization stage. Migration into these regions will increase their urban population growth rate to exceed that of wages. This will make the effect of the number of participants more dominant, which will have a significant positive impact on the solvency of their pension systems. However, soon these regions will transition into the late urbanization stage, the growth rate of the contribution base will gradually exceed that of the urban population. At this time, the effect of the contribution base will begin to dominate, which will harm the solvency of pension systems in these regions.

### Potential channel analysis

Based on the above theoretical proposition, we find that the impact of migration on pension solvency in migrant-receiving areas is mainly triggered by the number of participants and the contribution base. In this section, we test these two triggers.

Table 6 reports the test results of the number of participants and the contribution base. In Column (1)–(2), we find that there is a significantly positive correlation between migration and the number of participants. As the urbanization rate gradually increases, the effect of migration on pension solvency also increases. This observation is consistent with Han (2013) who investigated 14 European countries [33] and found that migration can effectively ease the pressure on public pension systems in countries with Bismarckian pension systems. In Column (3), we can see an inverted U-shape which indicates the association between migration and the contribution base. These two triggers have entirely different effects on the solvency of pension systems. Migration causes the solvency of pension systems in migrant-receiving areas to appear as an inverted-U curve at different urbanization stages.

**Table 6:** Potential channel analysis (FE estimation results).

Variables	Dependent variable: Pension solvency		
	Number of participants		Contribution base
	(1)	(2)	(3)
Migration rate	-0.882*	-0.878***	-7.877***
	(0.460)	(0.459)	(2.289)
Migration rate * Urbanization rate	1.848***	1.812**	16.333***
	(1.005)	(0.756)	(4.476)
Migration rate * Urbanization rate ^2^	-0.035		-5.402***
	(0.750)		(1.810)
Urbanization rate	-1.349	-1.343	-7.558***
	(0.807)	(0.822)	(3.893)
Other controls	✓	✓	✓
Provincial fixed effects	✓	✓	✓
Observations	527	527	527
Adj R^2^	0.816	0.817	0.982

Notes

***, **, and * indicate significance at 1%, 5%, and 10%, respectively. Robust standard errors with a cluster at the province level are presented in parentheses. Other controls include old-age dependency ratio, economic development level, pension benefits, medical insurance, and maternity insurance.

## Conclusion

Since the beginning of the 21st century, population aging has placed heavy pressure on China’s public pensions. The Chinese government is trying to alleviate population aging and reform the public pension systems through various measures, including adjusting the birth control policy and implementing the central pension transfer system. However, how can migration be of benefit to the solvency of the pension systems?

This paper investigates the relationship between migration and the solvency of pension systems in migrant-receiving areas, theoretically and practically. The results show that with the combined effect of the number of participants and the contribution base, the impact of migration on pension solvency is an inverted-U curve at different stages of urbanization. Further analysis by region shows that the inverted U-shape mainly appears in the central and western regions.

Our major findings are: First, to optimize the current pension system in China, local policies to improve the central pension transfer system without losing efficiency and fairness should be implemented. Second, subject to the transfer of the pension cost caused by migration, China needs to gradually adjust its pension system from the current Pay-As-You-Go to structure to a cumulative funding structure.

## Supporting information

S1 Fig Migration rate by province in 2018. (DOCX)

S2 Fig The relationship between migration and pension solvency at each stage of urbanization. (DOCX)

S3 Fig The trend of national and regional urbanization rate from 2002 to 2018. (DOCX)

S1 Table The balance of the public pension fund for urban employees. (DOCX)

S2 Table The balance of public pension fund by province in 2015. (DOCX)
