# Supplementary material for: Relationships between migration and the fiscal sustainability of the pension system in China
Source: PLoS One. 2021 Mar 10;16(3):e0248138. doi: 10.1371/journal.pone.0248138 (PMC7946295; doi:10.1371/journal.pone.0248138)
Supplement: S1 Fig — (DOCX) [file pone.0248138.s001.docx]

**

**

**S1 Fig. Migration rate by province in 2018.**

Note: migration rate = permanent population / registration population. The data of the permanent population comes from the National Bureau of Statistics, and the data of the registered population comes from *China’s Population and Employment Statistics Yearbook* in 2019. Above the dash line represents the net inflow areas, while below the dash line represents the net outflow areas.
