# Supplementary material for: Relationships between migration and the fiscal sustainability of the pension system in China
Source: PLoS One. 2021 Mar 10;16(3):e0248138. doi: 10.1371/journal.pone.0248138 (PMC7946295; doi:10.1371/journal.pone.0248138)
Supplement: S2 Fig — (DOCX) [file pone.0248138.s002.docx]

**S2 Fig. The relationship between migration and pension solvency at each stage of urbanization.**
