# Supplementary material for: Relationships between migration and the fiscal sustainability of the pension system in China
Source: PLoS One. 2021 Mar 10;16(3):e0248138. doi: 10.1371/journal.pone.0248138 (PMC7946295; doi:10.1371/journal.pone.0248138)
Supplement: S3 Fig — (DOCX) [file pone.0248138.s003.docx]

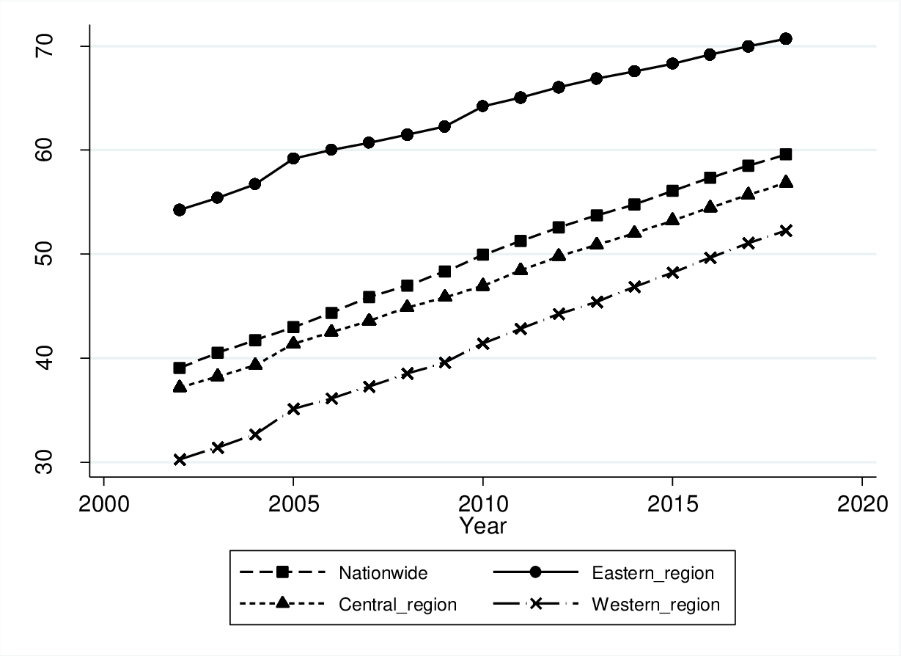


**S3 Fig. The trend of national and regional urbanization rate from 2002 to 2018.**

Note: The data is from the National Bureau of Statistics of China*,* 2002-2018*.*
