# Supplementary material for: Relationships between migration and the fiscal sustainability of the pension system in China
Source: PLoS One. 2021 Mar 10;16(3):e0248138. doi: 10.1371/journal.pone.0248138 (PMC7946295; doi:10.1371/journal.pone.0248138)
Supplement: S1 Table — (DOCX) [file pone.0248138.s004.docx]

**S1 Table. The balance of the public pension fund for urban employees.**

| Year | Total number of participants (million) | Total number of retirees (million) | Growth rate of total contribution (%) | Growth rate of total expenditure (%) | Growth rate of accumulative balance (%) |
| --- | --- | --- | --- | --- | --- |
| 2002 | 111.29 | 36.08 | 27.42 | 22.47 | 52.55 |
| 2003 | 116.46 | 38.60 | 16.03 | 9.82 | 37.22 |
| 2004 | 163.53 | 41.03 | 15.72 | 12.17 | 34.83 |
| 2005 | 174.87 | 43.67 | 19.61 | 15.37 | 35.83 |
| 2006 | 187.66 | 46.35 | 23.88 | 21.19 | 35.83 |
| 2007 | 201.37 | 49.54 | 24.16 | 21.82 | 34.66 |
| 2008 | 218.91 | 53.04 | 24.33 | 23.88 | 34.36 |
| 2009 | 235.50 | 58.07 | 17.97 | 20.36 | 26.13 |
| 2010 | 257.07 | 63.05 | 16.78 | 18.67 | 22.67 |
| 2011 | 283.91 | 68.26 | 25.90 | 20.94 | 26.89 |
| 2012 | 304.27 | 74.46 | 18.39 | 21.91 | 22.80 |
| 2013 | 322.18 | 80.41 | 13.40 | 18.69 | 18.08 |
| 2014 | 341.24 | 85.93 | 11.59 | 17.78 | 12.49 |
| 2015 | 353.61 | 91.42 | 15.93 | 18.65 | 11.15 |
| 2016 | 379.30 | 101.03 | 19.48 | 23.40 | 9.15 |
| 2017 | 402.93 | 110.26 | 23.54 | 19.46 | 13.75 |
| 2018 | 419.02 | 117.98 | 18.14 | 17.33 | 15.99 |
