# Supplementary material for: Relationships between migration and the fiscal sustainability of the pension system in China
Source: PLoS One. 2021 Mar 10;16(3):e0248138. doi: 10.1371/journal.pone.0248138 (PMC7946295; doi:10.1371/journal.pone.0248138)
Supplement: S2 Table — (DOCX) [file pone.0248138.s005.docx]

**S2 Table. The balance of public pension fund by province in 2015.**

| Province | Balance (100 Million Yuan) | Province | Balance (100 Million Yuan) | Province | Balance (100 Million Yuan) |
| --- | --- | --- | --- | --- | --- |
| Guangdong | 797.60 | Anhui | -34.26 | Shanghai | -203.62 |
| Beijing | 513.31 | Qinghai | -36.04 | Hunan | -213.57 |
| Zhejiang | 172.40 | Hainan | -49.95 | Henan | -233.70 |
| Jiangsu | 117.68 | Gansu | -79.97 | Jilin | -239.42 |
| Shandong | 30.05 | Jiangxi | -110.43 | Sichuan | -275.67 |
| Fujian | 19.10 | Tianjin | -124.47 | Hubei | -324.76 |
| Tibet | 3.69 | Shanxi | -132.96 | Hebei | -334.19 |
| Guizhou | -0.43 | Guangxi | -136.66 | Heilongjiang | -561.14 |
| Xinjiang | -3.90 | Chongqing | -160.84 | Liaoning | -692.98 |
| Ningxia | -27.05 | Inner Mongolia | -162.53 |  |  |
| Yunnan | -30.91 | Shaanxi | -171.02 |  |  |
